# Effectiveness and safety of massage in the treatment of anxiety and depression in patients with cancer: A protocol for systematic review and meta-analysis

**DOI:** 10.1097/MD.0000000000022262

**Published:** 2020-09-25

**Authors:** Siyu Qin, Yuanyi Xiao, Zhenhai Chi, Daocheng Zhu, Pan Cheng, Ting Yu, Haiyan Li, Lin Jiao

**Affiliations:** a College of Acupuncture-Moxibustion and Tuina, Jiangxi University of Traditional Chinese Medicine; b The Affiliated Hospital of Jiangxi University of Traditional Chinese Medicine, Nanchang, China.

**Keywords:** anxiety, cancer, depression, massage, protocol, systematic review

## Abstract

**Background:**

Anxiety and depression, complications of cancer, are prevalent but often overlooked mental illnesses. Studies have demonstrated that massage therapy is useful in relieving anxiety and depression of cancer survivors. However, the mechanism is still unclear and no systematic review has provided sufficient evidence for the treatment. Therefore, this protocol is carried out to comprehensively evaluate the reliability of cancer patients with anxiety and depression treated by massage.

**Methods:**

We will systematically search the relevant literature from PubMed, Cochrane Library, EMBASE, Web of Science, Wanfang, Chongqing VIP, CNKI and Chinese Biomedical Literature Database from the establishment of the databases to June 1, 2020. In addition, we will only include randomized controlled trials about massage for cancer survivors with anxiety and depression, regardless of language and publication status. Two experienced researchers will separately screen the literature, collect data, analyze data and synthesize data using RevMan V.5.3 software. The quality of the included trials in the study will be assessed by the Cochrane bias risk assessment tool.

**Results:**

The protocol for the meta-analysis will systematically evaluate the reliability of massage therapy for cancer patients with anxiety and depression.

**Conclusion:**

This conclusion will provide an important basis for evaluating whether massage is reliable in treating cancer survivors who feel anxious and depressed.

**INPLASY registration number:**

INPLASY202060101

## 1 Introduction

Cancer, a concerning public health problem, threatens the health of human beings all over the world. There are 18.1 million new cancer patients and 9.6 million cancer deaths in 2018.^[1]^ Not only the physical health of patients but also their mental health can be significantly affected by a diagnosis of cancer.^[2]^ Anxiety and depression, complications of cancer, are prevalent but often overlooked mental illnesses.^[3]^ Many research have also shown that the most common psychological states with cancer patients are anxiety and depression.^[4–9]^ Meanwhile, two-thirds of cancer survivors with depression are often associated with significant anxiety in the clinical.^[10]^ Currently, a series of studies have reported that anxiety and depression can produce some negative effects on patients’ quality of life (QOL), health expenditures, continuity of treatment, and cancer survival.^[3,11,12]^ Therefore, it is very important to find more effective treatments alleviating anxiety and depression symptoms of patients who have been diagnosed with cancer.

At present, pharmacotherapy and psychotherapy, the main treatment means for anxiety and depression of cancer survivors, play an important role in improving these distressing emotions.^[2,3,12,13]^ However, antidepressants and anxiolytics may bring a variety of adverse impacts such as headaches, addiction, seizures, suicide, and interactions with anticancer treatments.^[13–15]^ Besides, the psychological intervention available to patients is also limited due to the lack of providers and financial resources.^[16]^ So, it is extremely necessary to seek an alternative treatment that is effective, cheaper, and safer. It is reported that Complementary and Alternative Medicine interventions are used by more than half of cancer patients to relieve related symptoms.^[17]^ Nowadays, massage which is 1 of the most widely used complementary and alternative medicine therapies can not only relieve cancer-related symptoms but also bring physical and mental pleasure.^[18]^

Massage, defined as a method of manipulating body tissue by hand, has certain effects on the vessel, nerves, and muscle system of the body.^[19]^ Compared to pharmacotherapy and psychotherapy, massage has unique advantages because of its non-invasive, low-cost, and safety characteristics.^[20]^ According to a report, in North American medical centers, massage treatment as a supportive treatment is gradually available for cancer survivors to improve comfort level, lessen symptoms and related side effects.^[21]^ Many studies have found that massage can reduce muscle fatigue, improve blood flow, relax mood as well as relieve cancer symptoms such as anxiety, depression, pain, and nausea.^[20,22–24]^Moreover, the result which was published in the Journal of Clinical Oncology (JCO) also demonstrated that massage could relieve anxiety and depression of cancer survivors.^[25]^

To our knowledge, there is no recent systematic review discussing whether massage therapy is safe and effective in treating anxiety and depression symptoms of patients who have been diagnosed with cancer. Therefore, we perform this protocol to comprehensively assess the effect of massage for cancer patients who feel anxious and depressed.

## 2 Methods

### 2.1 Study registration

The registration number of this study is INPLASY202060101. We will strictly perform this protocol by following the Preferred Reporting Items for Systematic Reviews and Meta-Analyses Protocol (PRISMA-P) statement guidelines.^[26]^

### 2.2 Inclusion criteria for study selection

#### 2.2.1 Type of studies

Only randomized controlled trials (RCTs) about massage for cancer survivors with anxiety and depression will be included. There are no restrictions on language and publication status. In addition, non-RCTs, experience report, reviews will not be included.

#### 2.2.2 Types of Participants

All cancer patients feeling anxious and depressed will be included. There are no limitations for age, ethnicity, and gender.

#### 2.2.3 Types of interventions

##### 2.2.3.1 Experimental interventions

The experimental group will only include massage therapies, which include reflexology, acupressure, manual lymphatic drainage, tuina, general massage, etc. There is no restriction on the types of massage, treatment sites, treatment time, and the frequency.

##### 2.2.3.2 Control interventions

The interventions of the control group will include any therapies without massage, such as drugs, psychotherapy, routine care, placebo, cupping therapy, acupuncture, etc.

#### 2.2.4 Types of outcome measures

##### 2.2.4.1 Primary outcomes

1. The State Anxiety Inventory.
2. The Center for Epidemiological Studies Depression scale.

##### 2.2.4.2 Additional outcomes

1. The Quality of Life Questionnaire Core 30 from the European Organization for Research on Treatment of Cancer.
2. Any adverse events.

### 2.3 Search methods

RCTs relating to massage management for anxiety and depression of cancer survivors will be retrieved from PubMed, Cochrane Central Register of Controlled Trials, EMBASE, Web of Science, Chinese Biomedical Literature Database, Wanfang Database, Chongqing VIP Database and Chinese National Knowledge Infrastructure from the establishment of the databases to June 1, 2020. In addition, The Clinicaltrials.gov, Chinese Clinical Trial Registry will also be carefully retrieved to obtain unpublished or ongoing trial data. The detailed PubMed searching strategy is listed in Table 1.

**Table 1:**
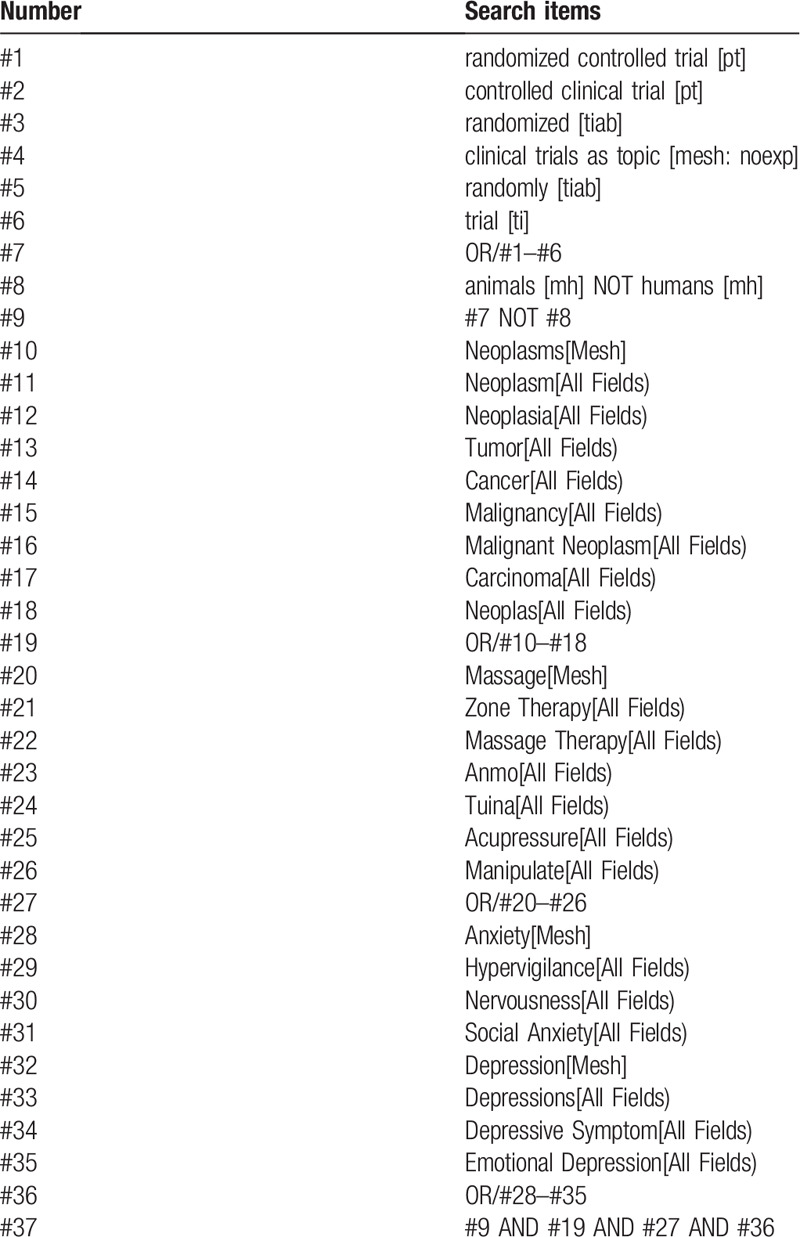
Search strategy used in PubMed database.

### 2.4 Data collection and analysis

#### 2.4.1 Selection of studies

**Figure 1:**
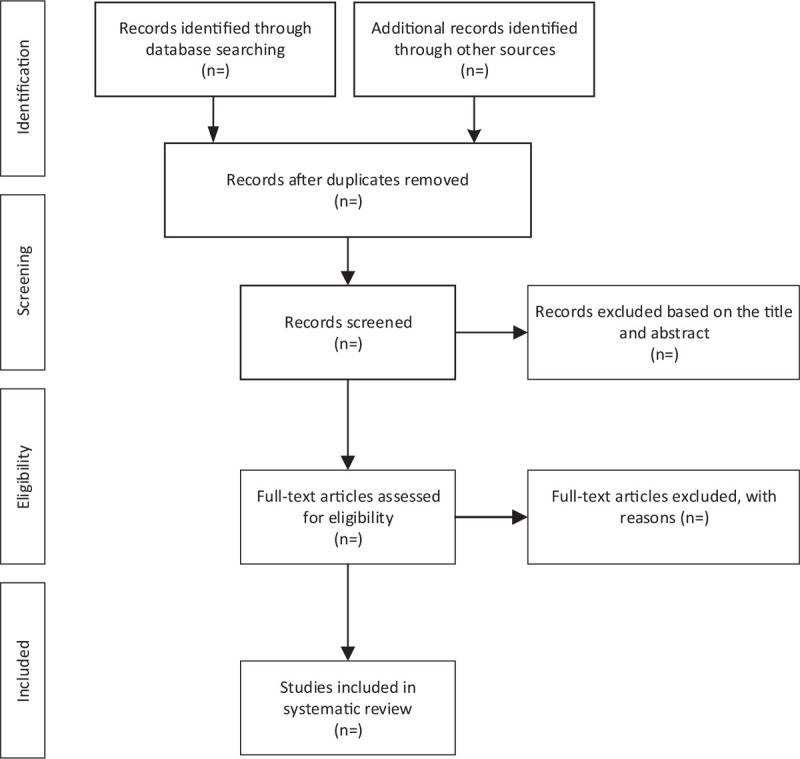
Flow diagram of study selection process.

All searched literature will be imported into EndNote software (V.X9) for removing duplicate literature. The 2 researchers (SQ and YX) will independently read the title and abstract to exclude irrelevant literature. After preliminary screening, they will carefully read the full text to determine whether the related studies are eventually included in the protocol. Then, a cross-check will be conducted by 2 researchers (SQ and YX). Finally, if there is any disagreement when the 2 researchers perform the above operation, it will be discussed or resolved by the third researcher (LJ). The specific literature screening flow chart will be presented in Figure 1.

#### 2.4.2 Data extraction and management

Two researchers (SQ and ZC) will separately extract the following informations by using data extraction forms which have been prepared in advance:

(1) Research Characteristics: Year of publication, Journal, title, information of the author.
(2) Participants’ basic information: Gender, age, course of the disease, country, sample size, severity of anxiety, and depression.
(3) Study methods: Randomization, allocation concealment, blinding, result analysis method.
(4) Intervention: Operation name, treatment sites, treatment time, course of treatment, and frequency.
(5) Outcomes measurement: Primary and secondary outcomes.

### 2.5 Evaluation of bias risk in included studies

Two experienced researchers (SQ and ZC) will separately assess the quality of the trials using the Cochrane bias risk assessment tool.^[27]^ It includes 7 aspects: random sequence generation, allocation concealment, blinding of participants and personnel, blinding of outcome assessment, incomplete outcome data, selective outcome reporting, and other bias. Each item can be classified as high, low, and unclear risk bias levels. When the items related to studies are not clear, we will contact the authors to get the required information. It is necessary to consult the third researcher (LJ) to make a reliable decision if there is any controversy.

### 2.6 Data synthesis

The following data analysis will be performed using RevMan 5.3 software. When the measured outcomes are dichotomous data, the risk ratio (RR) with 95% confidence interval (CI) will be adopted. When the measured outcomes are continuous data, Weighted Mean Difference (WMD) with 95% CI will be adopted if we use the same measurement instrument. And the Standardized Mean Difference (SMD) with 95% CI will be applied if we use different measurement instruments. *χ*^2^ test and *I*^2^ test will be utilized to investigate the heterogeneity. When the heterogeneity is considered to be not obvious (*P* > 0.1 or *I*^2^ < 50%), we will choose the fixed-effect model. On the contrary, when the heterogeneity is considered to be obvious (*P* ≤ 0.1 or *I*^2^ ≥ 50%), the random-effect model will be chosen and the subgroup analysis or sensitivity analysis will be conducted to seek potential causes of intergroup heterogeneity. A descriptive analysis is necessary to be carried out if the heterogeneity is too significant.

### 2.7 Management of missing data

If the data of the included studies are unclear or missing, we will do our best to contact the related authors of the article to acquire complete data. If the above way of obtaining data information is unsuccessful, we will only use current data for the data analysis.

### 2.8 Subgroup analysis

If significant heterogeneity exists in the included trials, it is necessary to perform a subgroup analysis to reduce heterogeneity between groups based on differences in massage methods, course of the disease, sample size, the severity of anxiety and depression, and so on.

### 2.9 Sensitivity analysis

If necessary, sensitivity analysis will be performed to evaluate whether the conclusions of the meta-analysis are stable or reliable by excluding trials of low quality.

### 2.10 Assessment of reporting biases

Funnel plots will be adopted to detect the publication bias if the included trials exceed 10. Egger test will be performed to analyze the potential causes of publishing bias if the asymmetry exists in the funnel plots.

### 2.11 Quality of evidence

The quality of evidence will be independently evaluated by 2 researchers using the Grading of Recommendations Assessment, Development, and Evaluation (GRADE),^[28]^ and will be assessed into high, moderate, low, and very low levels.

### 2.12 Ethics and dissemination

In the study, patients’ personal information is not involved, so ethical approval is not necessary. Results from this protocol will be disseminated in a peer-reviewed journal.

## 3 Discussion

Anxiety and depression, complications of cancer, seriously affect the mental health of cancer patients. Even though drug therapy and psychotherapy are effective in relieving anxiety and depression, these treatments may have some side effects. Massage as a complementary and alternative therapy has been widely used to alleviate anxiety and depression symptoms of patients who have been diagnosed with cancer, due to its non-invasive, safe, and inexpensive features.^[20]^ Studies have shown that massage therapy can be effective in easing mood and reducing cancer-related symptoms, including depression, anxiety pain, fatigue, and so on.^[20,24,25]^ However, its clinical mechanism of action is still unclear and no systematic review has provided sufficient evidence for this treatment. We hope that the results of this study are useful to patients, clinicians, and health policymakers.

However, the study may have the following limitations: First, significant heterogeneity may exist due to different massage methods, different treatment sites, and time. Second, certain language bias may be caused due to the absence of language limitations.

## Author contributions

**Conceptualization**: Siyu Qin, Yuanyi Xiao.

**Data curation**: Siyu Qin, Zhenhai Chi.

**Formal analysis**: Siyu Qin, Yuanyi Xiao.

**Funding acquisition**: Lin Jiao.

**Methodology**: Siyu Qin, Zhenhai Chi.

**Software**: Siyu Qin, Yuanyi Xiao.

**Supervision**: Lin Jiao.

**Writing – original draft**: Siyu Qin, Yuanyi Xiao.

**Writing – review & editing**: Lin Jiao.
